# The Association Between Selected Molecular Biomarkers and Ambulatory Blood Pressure Patterns in African Chronic Kidney Disease and Hypertensive Patients Compared With Normotensive Controls: Protocol for a Longitudinal Study

**DOI:** 10.2196/14820

**Published:** 2020-01-17

**Authors:** Abiodun Moshood Adeoye, Oladimeji Adebayo, Busayo Abiola, Bamidele Iwalokun, Bamidele Tayo, Fadi Charchar, Akinlolu Ojo, Richard Cooper

**Affiliations:** 1 Department of Medicine University College Hospital Ibadan Nigeria; 2 Department of Medicine, Faculty of Clinical Science College of Medicine University of Ibadan Ibadan Nigeria; 3 Division of Molecular Biology and Biotechnology Nigerian Institute of Medical Research Lagos Nigeria; 4 Public Health Sciences Stritch School of Medicine Loyola University Chicago Maywood, IL United States; 5 Faculty of Health and Life Sciences Federation University Australia Ballarat Australia; 6 Department of Medicine College of Medicine University of Kansas School of Medicine Kansas, KS United States

**Keywords:** chronic kidney disease, cardiovascular disease, ambulatory blood pressure

## Abstract

**Background:**

Chronic kidney disease (CKD) is a burgeoning epidemic in sub-Saharan Africa. Abnormal blood pressure variations are prevalent in CKD and potentiate the risk of cardiovascular morbidity and mortality. Certain genetic variants (angiotensin II receptor type 1 1166 A>C and angiotensin-converting enzyme insertion and deletion polymorphisms) and biomarkers such as interleukin–6, tumor necrosis factor, soluble (s) E-selectin, homocysteine, and highly sensitive C-reactive protein have been shown to affect blood pressure variability among non-African CKD, hypertensive. and nonhypertensive CKD population. However, the contributions of the pattern, genetic, and environmental determinants of ambulatory blood pressure in African CKD have not been characterized. Understanding these interactions may help to develop interventions to prevent major cardiovascular events among people with CKD.

**Objective:**

The overarching objective of this study is to identify, document, and develop approaches to address related phenomic, genetic, and environmental determinants of ambulatory blood pressure patterns in African CKD and non-CKD hypertensive patients compared with normotensive controls.

**Methods:**

This is a longitudinal short-term follow-up study of 200 adult subjects with CKD and 200 each of age-matched hypertensives without CKD and apparently healthy controls. Demographic information, detailed clinical profile, electrocardiography, echocardiography, and 24-hr ambulatory blood pressure measurements will be obtained. Blood samples will be collected to determine albumin-creatinine ratio, fasting plasma glucose, lipid profile, electrolytes, urea and creatinine, C-reactive protein, serum homocysteine, fibroblast growth factor–23, and complete blood count, while 2 mL blood aliquot will be collected in EDTA (ethylenediaminetetraacetic acid) tubes and mixed using an electronic rolling system to prevent blood clots and subsequently used for DNA extraction and genetic analysis.

**Results:**

A total of 239 participants have been recruited so far, and it is expected that the recruitment phase will be complete in June 2020. The follow-up phase will continue with data analysis and publications of results.

**Conclusions:**

This study will help stratify Nigerian CKD patients phenotypically and genotypically in terms of their blood pressure variations with implications for targeted interventions and timing of medications to improve prognosis.

**International Registered Report Identifier (IRRID):**

DERR1-10.2196/14820

## Introduction

### Background

Chronic kidney disease (CKD) is rising in public health importance in Nigeria and contributes significantly to the increased burden of cardiovascular disease (CVD) in the country and sub-Saharan Africa in general [1-4]. It has been estimated that approximately 36 million people are currently living with CKD in Nigeria with the most common causes being glomerulonephritis and hypertension [4,5]. The disease is associated with high morbidity and mortality and exorbitant cost of treatment [6,7]. Most patients requiring renal replacement therapy are unable to afford the cost of maintenance dialysis or kidney transplantation [8].

Poorly managed CKD is often associated with rapid progression to end-stage renal disease (ESRD) and cardiovascular disease (CVD) [9,10]. The rapidity of progression is especially higher among individuals of African descent [9]. Most deaths from CKD arise from cardiovascular complications, among which are heart failure, coronary artery disease, arrhythmia, and stroke [11]. In addition, many of the patients often present late, thus making early detection and cardiovascular risk assessment among patients with CKD challenging. Efforts to improve cardiovascular outcomes in CKD in sub-Saharan Africa require an improved risk stratification scheme and early detection so that resources can be targeted at those CKD patients who are at increased risk of CVD events.

### Association Between Selected Molecular Biomarkers and Ambulatory Blood Pressure Pattern

CKD’s contribution to CVD is through many pathophysiologic mechanisms, cardinal among which is the failure of nocturnal blood pressure (BP) dipping. Studies have shown that being a nondipper increases the risk of CVD by 2.4 fold in patients with CKD [12-14]. Furthermore, nondipping of BP has been shown to increase susceptibility to CKD progression to ESRD, CVD, and poor responses to antihypertensive medications despite optimal compliance to treatment [12,14].

Previous studies have identified various biomarkers in non-African non-CKD hypertensive blacks to be associated with BP variability, including interleukin–6, tumor necrosis factor–alpha, soluble (s) E-selectin, homocysteine, and high-sensitivity C-reactive protein [15-18]. Some of these biomarkers are also linked to CVD adverse events in apparently healthy individuals [19].

### Renin-Angiotensin-Aldosterone Pathway Genetic Polymorphisms in Circadian Blood Pressure Variations

The current approach to reducing the CVD burden is chronotherapy, through which 24-hour optimal BP control is ensured. However, not all patients respond well to chronotherapy, suggesting that the mechanism underlying the nondipping of BP and nocturnal hypertension in CKD is not completely understood [12,20]. Studies in non-Africans have suggested that the genetic variants in the renin-angiotensin-aldosterone pathway may play a critical role in the nondipping of BP, and the two genetic variants implicated are the angiotensin II type 1 receptor 1166 adenine to cytosine (AGTR1 1166 A>C) polymorphism and the insertion/deletion mutant of the angiotensin converting enzyme (ACE I/D) gene [21,22].

The mutations in the two genes have been shown to cause abnormal activation of the renin-angiotensin-aldosterone system (RAAS) with characteristic elevations in the systemic levels of renin, aldosterone, and angiotensin II, which eventually lead to potassium ion loss and sodium ion retention, left ventricular hypertrophy, and atrial fibrillation [22,23]. Abnormally activated RAAS also leads to vasoconstriction of the arteries including the coronary artery and other vessels, inducing cellular proliferation of these vasculatures and stimulating extracellular matrix protein synthesis [24]. Both are required for fibrotic changes in the heart muscle and kidney and blood vessels [24]. The angiotensin II receptor and ACE mediate RAAS pathophysiological activities through two distinct functional polymorphisms of their genes. These polymorphisms are AGTR1 1166 A>C and the ACE I/D gene [23,24].

This study seeks to identify, document, and develop approaches to address related phenomic, genetic, and environmental determinants of ambulatory BP patterns in African CKD and non-CKD hypertensive patients compared with normotensive controls. We will also explore the associations between genetic variants in the RAAS pathway and nocturnal BP variations and its cardiovascular sequelae among CKD patients.

### Aims

1. Evaluate the contributions of traditional and novel sociodemographic, clinical, and environmental risk factors to ambulatory BP patterns in African CKD and hypertensive patients compared with normotensive controls
2. Determine AGTR1 1166 A>C and ACE I/D genotypes and allele distributions among nondipper Nigerians with CKD and nonhypertensive CKD while comparing the same among dippers and nondippers
3. Determine the association between AGTR1 1166 A>C and ACE I/D genotypes and ambulatory BP phenotypes (diurnal BP variability)
4. Determine the association between AGTR1 1166 A>C and ACE I/D genotypes and cardiovascular risk factors (hyperuricemia, hyperglycemia or diabetes, hypertriglyceridemia) among Nigerians with CKD and nonhypertensive CKD
5. Determine the association between AGTR1 1166 A>C and ACE I/D genotypes and major cardiovascular events (stroke, myocardial infarction, heart failure, atrial fibrillation, and abnormal left ventricular geometry) among Nigerians with CKD and nonhypertensive CKD
6. Determine the genes by environmental interactions that are associated with nocturnal BP variability among Nigerians with CKD

### Hypotheses

1. Unique environmental risk factors will contribute to ambulatory BP pattern in African CKD and hypertensive individuals of African ancestry.
2. Polymorphisms of AGTR1 1166 A>C and ACE I/D are higher among CKD dippers compared with CKD nondippers.
3. Polymorphisms of AGTR1 1166 A>C and ACE I/D are associated with ambulatory BP phenotypes and cardiovascular risk factors in CKD and non-CKD hypertensives.
4. Polymorphisms of AGTR1 1166 A>C and ACE I/D are associated with cardiovascular risk factors among patients with CKD.
5. Polymorphisms of AGTR1 1166 A>C and ACE I/D do not interact with environmental factors (body mass index, cigarette smoking, alcohol intake, and medication adherence) in the development of nondipping.

## Methods

### Design

This is a longitudinal short-term follow-up study of 200 adult subjects with CKD and 200 each of age-matched hypertensives without CKD and apparently healthy controls. The CKD cases and non-CKD hypertensive patients will be recruited from the medical outpatient clinics at the University College Hospital, Ibadan, and the Nigerian Institute of Medical Research (NIMR) [25], Yaba, Lagos. Controls will be recruited during medical outreaches organized in communities from which CKD patient referrals are received.

### Ethics Approval and Consent to Participate

The study was approved by the University of Ibadan/University College Hospital Research Ethical Committee with approval number UI/EC/18/0291. Participants are required to give written informed consent to participate in the study.

### Study Sites

The study will be carried out in the NIMR and the Department of Medicine, University of Ibadan University College Hospital, a teaching hospital with a 850-bed capacity and several community care centers in Ibadan and other sites within the state from where CKD cases are referred. It has full complements of all clinical and nonclinical departments. The hospital provides both preventive and curative services and is equipped with cardiac, neuroimaging, and other laboratory facilities. From the registry, a monthly average of 50 cases of CKD are seen in the renal clinic.

NIMR is the hub for research, human resource capacity building, and collaboration for national development in the country. It is a foremost health research institute established in 1977 to conduct basic, applied, and implementation science research on infectious and noncommunicable diseases of public health importance in Nigeria. NIMR is made up of five major divisions: Microbiology, Public Health, Biochemistry and Nutrition, Molecular Biology and Biotechnology, and Clinical Sciences and has three support research units: Centre for Human Virology and Genomics, Centre for Tuberculosis Research, and Centre for Alternative Medicine and Research.

### Inclusion and Exclusion Criteria

#### Chronic Kidney Disease Participants

The inclusion criteria for the study are being aged ≥18 years and having a diagnosis of CKD, while the exclusion criteria are diagnosis of autosomal polycystic kidney disease or sickle cell nephropathy (Textbox 1).

**Variables and definitions.**

- Chronic kidney disease: estimated glomerular filtration rate less than 60 mL/min/1.73 m2 on two or more occasions at least 3 months apart
- Hypertension: systolic blood pressure ≥140 mm Hg, diastolic blood pressure ≥90 mm Hg or both and on antihypertensive medication
- Diabetes mellitus: fasting blood sugar ≥126 mg/dL or on medications for diabetes
- Waist circumference: >102 cm (male) and >88 cm (female)
- Obese: body mass index ≥30 kg/m2
- Dyslipidemia: total cholesterol >200 mg/dL (male/female), high-density lipoprotein <40 mg/dL (male), <50 mg/dL (female), or low-density lipoprotein >130 mg/dL (male/female)
- Hyperuricemia: serum uric acid >7 mg/dL (male) and >6 mg/dL (female)

#### Hypertensive Participants

The inclusion criteria for the study are being aged ≥18 years and having a diagnosis of hypertension, while the exclusion criteria are diagnosis of autosomal polycystic kidney disease, sickle cell nephropathy, or CKD (Textbox 1).

#### Controls

The inclusion criteria for the study are being aged ≥18 years and apparently clinically normal, while the exclusion criteria are subjects with autosomal polycystic kidney disease, sickle cell nephropathy, or CKD.

### Evaluation of Cases and Controls

The study will be in 2 phases. In the initial phase, data will be collected in standard case report form (available on request) and information regarding bio-data, risk factors for CVD, lifestyle, stage of CKD, and comorbidities will be obtained. Other important variables will include clinical features and medication use of participants.

Demographic information, detailed clinical profile, electrocardiography, echocardiography, and 24-hour ABPM will be obtained. In addition, the dialysis vintage, frequency of comorbidities, family history of hypertension and kidney disease, medication, and medication adherence using pill count will be accessed.

### Follow-Up Strategy

Participants will subsequently be assessed at 6 months, 1 year, and 2 years for any cardiovascular adverse outcomes. Major adverse cardiovascular events such as cardiac death and hospitalization for cardiac events will be assessed while noncardiovascular death and events will also be noted. Serum creatinine will be assessed.

### Anthropometric Measurements

Anthropometric measurements (height, weight, and hip and waist circumferences) will be performed using World Health Organization guidelines [26,27]. A semiautomated digital BP machine will be used to measure BP in accordance with World Health Organization guidelines, and the average readings will be recorded.

### 24-Hour Ambulatory Blood Pressure Monitoring

24-hour ABPM will be done using Spacelabs 90207 monitors (Spacelabs Healthcare) which will be placed on the nondominant hand within the first week of recruitment. The machine will be programmed to read every half hour from 7:00 am to 10:00 pm and hourly from 10:00 pm to 7:00 am during weekdays. Patients will be encouraged to proceed with their routine daily activities but avoid strenuous physical activities and keep motionless at the time of measurement. Dipping pattern, white coat effects, and nocturnal and daytime hypertensions will be deduced from the readings.

### Body Fluids Sampling Strategy

Venous blood samples (5 mL) will be drawn from each study participant after an overnight fast by medical personnel with phlebotomy training at each site. All tests and examinations will be performed between 8:00 am and 10:00 am daily. Three mLs aliquot of the blood sample will be collected in plain tubes to determine albumin-creatinine ratio, fasting plasma glucose, lipid profile, electrolytes, urea and creatinine, C-reactive protein, serum homocysteine, fibroblast growth factor–23, and complete blood count, while the remaining 2 mL aliquot will be collected in EDTA (ethylenediaminetetraacetic acid) tubes and mixed using an electronic rolling system to prevent blood clots and subsequently used for DNA extraction and genetic analysis.

Early morning urine and fasting blood sample will be collected. Estimated glomerular filtration rate (eGFR) will be calculated using the Modification of Diet in Renal Disease 4-variable equation. Subjects will be followed up at 3-month intervals for 1 year.

### Genetic Analysis

#### Angiotensin Converting Enzyme Gene II/ID/DD Polymorphism

QIAamp DNA Blood Mini Kits (Qiagen) will be used to extract DNA from blood cells. A 287-bp I/D polymorphism in the intron 16 of the ACE gene will be examined by polymerase chain reaction (PCR). The sequences of the sense and antisense primers were 5′-CTG GAG ACC ACT CTT TCT-3′ and 5′-GAT GTG GCC ATC ACA TTC GTC AGA-3′, respectively. Both forward and reverse primers will be synthesized by Inqaba Biotec. The PCR will be performed in a final volume of 20 μL, containing 100 ng of genomic DNA, 40 pmol of each primer, 200 μmol each of the four dNTP, 3 mM MgCl_2_, 50 mmol KCl, 10 mmol Tris-HCl (pH 8.3), and 1.5 U of Taq polymerase (Promega Corp). Amplification will be carried out for 30 cycles with steps of denaturation at 94°C for 1 minute, annealing at 54°C for 1 minute, and extension at 72°C for 1 minute. The PCR products were subjected to electrophoresis in 1% agarose gels. Amplification of the D allele resulted in a 190-bp DNA fragment and amplification of the I allele resulted in a 490-bp fragment. Homozygotes had a single 190 (DD)– or 490 (ID)–bp band; heterozygotes had one 190-bp and one 490-bp band. The PCR products will be analyzed on 2% agarose gels with a 100-bp DNA Ladder (Promega Corp), and images were acquired and analyzed using a gel imaging system.

#### Angiotensin II Type 1 Receptor 1166 Adenine to Cytosine Polymorphism

The AGTR1 gene will be genotyped for 1166 A>C polymorphism by polymerase chain reaction-restriction fragment length polymorphism (PCR-RFLP) method as described by Zhu et al [20] using the following primers: 5′AATGCTTGTAGCCAAAGTCACCT and 5′ GGCTTTGCTTTGTCTTGTTG. PCR amplification will be performed in a 20 µl reaction containing 100 ng of genomic DNA, 1.5 mM MgCl_2_, 200 µM each of the deoxynucleotide triphosphates, 10 picomoles each of the forward and reverse primers, and 1.5 U of Taq DNA polymerase. PCR amplification will then be carried out in the SimpliAmp Thermal Cycler (Applied Biosystems). The conditions for PCR amplification consisted of 2 minutes denaturation at 94°C, followed by 40 cycles of 1 minute at 94°C, 1 minute annealing at 60°C, extension for 2 minutes at 72°C, and final extension for 10 minutes at 72°C. The resulting PCR products of the expected size (850-bp) will subsequently be analyzed by agarose gel electrophoresis with 1% agarose gel prestained with 0.5 µg/mL ethidium bromide in Tris-borate-EDTA buffer (pH 8.3) at 70 volts for 1 hour. To characterize the polymorphism, PCR products will be digested overnight with the restriction endonuclease Dde I (BioLabs Inc) at 37°C. This is expected to cut the product into two pieces, 600-bp and 250-bp long. An additional Dde I recognition site will be created in the C-type variant at nucleotide 1166, which is located within the 250-bp fragment. Therefore, the homozygote AA is expected to produce two bands (600-bp and 250-bp long), heterozygote AC to produce four bands (600-bp, 250-bp, 140-bp, and 110-bp long), and homozygote CC to produce three bands (600-bp, 160-bp, and 110-bp long). Analysis of the digested PCR products will also be done by agarose gel electrophoresis by using 3% agarose gel prestained with ethidium bromide in Tris-borate-EDTA buffer (pH 8.3), using gel electrophoresis apparatus (Promega Corp) and ultraviolet transillumination (Applied Biosystems). All the PCR products will be analyzed with a 100-bp DNA Ladder (Promega Corp), and gel images of DNA bands will be acquired and analyzed using a gel imaging system.

### Outcomes

The primary outcomes will be a composite of cardiovascular events (myocardial infarction, heart failure, and stroke) requiring hospitalization and death. Secondary outcomes will be a composite of ESRD, 50% decline in eGFR, and doubling of serum creatinine. Composite of hospitalization from cardiovascular events such as myocardial infarction, heart failure, and stroke will be significantly higher among CKD subjects compared with controls.

### Statistical Analysis and Power Calculation

The statistical analysis will be carried out using R (The R Foundation), and all categorical variables will be compared by chi-square test or Fisher exact test where indicated. Continuous variables that are normally distributed will be compared by means of the Student *t* test and analysis of variance where there are more than 2 groups. Continuous variables not normally distributed will be compared by the Mann-Whitney U test or the Kruskal-Wallis test where there are more than 2 groups. Correlations between two continuous variables will be assessed using Pearson correlation statistics. Linear and multiple logistic regression analyses will be used to determine factors predicting nondipping of BP and cardiovascular sequelae. The allele frequencies of the candidate gene variants will be determined for the AGTR1 1166 A>C and ACE I/D in CKD nondipper and controls. A test for associations between the variants and BP phenotypes will be performed by fitting covariate-adjusted logistic regression models in which each variant will be presented as a predictor variable whose values are equal to the number of copies of the minor allele (0, 1, 2; ie, additive mode) or presence of at least one copy of the minor allele (0, 1; ie, dominant mode) or presence of two copies of the minor allele (0, 1; ie, recessive mode). All tests of association will be adjusted for gender, age (to account for possible residual effects due to incomplete matching), severity of CKD, and other relevant covariates. Both ACE DD/ID and AGTR1 1166 A>C polymorphisms will also be evaluated for Hardy-Weinberg Equilibrium in the control group. The logistic model can be represented as logit[(pr=D)] = α + β1G + β2Sex+ β3Age (D denotes diurnal BP variation status, G denotes genetic variants coded as additive or dominant or recessive, β denotes the corresponding coefficient for each variable in the model [single-nucleotide polymorphisms, sex, or age] and its exponential is the corresponding odds ratio). Statistical significance for the adjusted odds ratio will be set at 0.05.

The power analysis for the genetic analysis is based on the primary analysis to assess the main effects of AGTR1 1166 A>C and ACE I/D polymorphisms and their interaction with environmental factors in Nigerians with CKD. Based on minimum allele frequencies of 0.12 and 2×105 for AGTR1 1166 A>C and ACE I/D polymorphisms, respectively. We will also assume that three genotypes of AGTR1 1166 A>C are equally distributed, and the probability of developing nondipping in the reference genotype group is 0.3. Data will be analyzed using SPSS version 20 (IBM Corp). Given a sample size of 600 (200 per group), this study design would have at least 80% power to detect a minimum odds ratio of 2.0 in patients with any of the three gene variants, using Bonferroni correction with type I error of 5%.

## Results

The study is supported with an International Society of Hypertension (ISH) Research Scholar Grant Award. A total of 239 participants have been recruited so far, and it is expected that the recruitment phase will be complete in June 2020. The follow-up phase will continue with data analysis and publications of results.

## Discussion

The prevalence of CKD and the associated adverse outcomes are on the increase in Nigeria [4,5,9,10]. These come with enormous socioeconomic implications, most especially in the light of inadequate understanding of BP variability among CKD patients through which appropriate strategies for treatment and prevention can be developed. It is therefore imperative to study the phenomics, genetics, and environmental determinants of ambulatory BP patterns in black CKD and non-CKD hypertensives.

There is evidence of association of AGTR1 1166 A>C and ACE I/D polymorphisms with CKD and cardiovascular diseases, their roles in non-BP dipping at night, and nocturnal hypertension, but this remain unclear among African blacks. The effects of these polymorphisms on Nigerian CKD patients either in the context of BP regulation or response to antihypertensive medications and other cardiovascular risk factors (hyperuricemia, hyperglycemia or diabetes, hypertriglyceridemia) have not been evaluated.

Given the fact that genetic environment influences the pathogenesis of CKD, cardiovascular risk factors, and cardiovascular diseases, there is a need to stratify Nigerian CKD patients genotypically using a translational research approach prior to integrating supportive therapy of CKD management for improved prognosis. Unraveling the role of these genetic polymorphisms among nondipper Nigerian patients with CKD will provide insight into the mechanisms underlying nondipping and CVD complications. This will also guide appropriate stratification into risk of ESRD progression and CVD risk profiling for adequate treatment plans, prevention plans, and appropriate policy changes.

Furthermore, identification of CKD patients at risk of nondipping using molecular and genetic markers will guide in appropriate use of cardiovascular risk stratification and predicts response to chronotherapy.

This case control study of 600 participants with 3 arms of 200 each, comprising CKD nondippers, CKD dippers, and apparently healthy controls will use comprehensive phenotyping and genotyping of RAAS pathway variants to identify genetic risk markers among CKD nondippers. It will be the first major study to assess this among African blacks.

This study will also explore the potential of the use of PCR-RFLP as a laboratory test for CKD with hypertension prior to treatment initiation for better stratification of patients for morning or night antihypertension medications in Nigeria. This innovative tool is rapid, cost effective, and highly applicable to larger cohorts of CKD patients compared with the more laborious, time-consuming conventional ABPM required for the identification of nondippers and patients with nocturnal hypertension. The PCR-RFLP technique will become a routine point-of-care test for personalized treatment of hypertension among affected CKD patients in the country.

## Appendix

Multimedia Appendix 1 Peer-reviewer report 1 from the University of Ibadan/University College Hospital Research Ethical Committee.

Multimedia Appendix 2 Peer-reviewer report 2 from the University of Ibadan/University College Hospital Research Ethical Committee.

## Abbreviations

ABPM: ambulatory blood pressure monitoring
ACE: angiotensin-converting enzyme
ACE I/D: insertion/deletion mutant of the angiotensin converting enzyme
AGTR1: angiotensin II type 1 receptor
AGTR1 1166 A>C: angiotensin II type 1 receptor 1166 adenine to cytosine
BP: blood pressure
CKD: chronic kidney disease
CVD: cardiovascular disease
EDTA: ethylenediaminetetraacetic acid
eGFR: estimated glomerular filtration rate
ESRD: end-stage renal disease
ISH: International Society of Hypertension
NIMR: Nigerian Institute of Medical Research
PCR: polymerase chain reaction
PCR-RFLP: polymerase chain reaction–restriction fragment length polymorphism
RAAS: renin-angiotensin-aldosterone system
