# Supplementary material for: The Association Between Selected Molecular Biomarkers and Ambulatory Blood Pressure Patterns in African Chronic Kidney Disease and Hypertensive Patients Compared With Normotensive Controls: Protocol for a Longitudinal Study
Source: JMIR Res Protoc. 2020 Jan 17;9(1):e14820. doi: 10.2196/14820 (PMC6996765; doi:10.2196/14820)
Supplement: Multimedia Appendix 1 [file resprot_v9i1e14820_app1.pdf]

November 23, 2018

The Chairperson,

UI/UCH Ethics Committee

IAMRAT, College of Medicine, University of Ibadan

Dear Ma,

Re: Association between Selected Molecular Biomarkers And Ambulatory Blood Pressure Pattern In African Chronic Kidney Disease And Hypertensive Patients Compared With Normotensive Controls

1. Thank you for asking me to review the above -titled proposal.

2. The proposal presents an original idea that has potentials to contribute to our understanding of the chronobiology of blood pressure in an indigenous Black African ancestry population.

3. The case - control design is appropriate. However, the sampling strategy needs further clarification. The PI described recruitment of consecutive cases that present to the clinic and community -based controls. How will this sampling approach work in the community ?

4. What is the total volume of blood to be collected from each study participant ? Will the subjects be bled once or at multiple times ? There seems to be discrepancy between volumes stated in the research strategy and the consent form.

5. Who bears the cost of the investigations including the genetic testing ?

6. I consider other aspects of the proposal appropriate and without any ethical, legal or sociological issues.

7. The proposal may be approved executively once the PI has satisfactorily addressed the minor issues raised.

Thank you for the opportunity to be of service to the ethics committee.
