# Supplementary material for: The Association Between Selected Molecular Biomarkers and Ambulatory Blood Pressure Patterns in African Chronic Kidney Disease and Hypertensive Patients Compared With Normotensive Controls: Protocol for a Longitudinal Study
Source: JMIR Res Protoc. 2020 Jan 17;9(1):e14820. doi: 10.2196/14820 (PMC6996765; doi:10.2196/14820)
Supplement: Multimedia Appendix 2 [file resprot_v9i1e14820_app2.pdf]

Report on Proposal for Ethical Review: Association between Selected Molecular Biomarkers and Ambulators Blood Pressure Pattern in Africa Chronic Kidney Disease and Hypertensive Patients Compared with Normotensive Controls.

Comments:

1. **Study Title:** Adequately reflect the subject being studied.
2. **Background to the study**- Satisfactory presented.
3. **Justification:** Not presented. In a few lines the author(s) should justify the need for that study in Nigeria.
4. **Objectives of the study:** Clearly stated under methods/Design.
5. **Research Design:** A longitudinal follow-up study with consecutive sampling method. This is satisfactory.

There is confusion in nominative of subjects and controls under inclusion/exclusion criteria on pages 5 & 6. The subjects and the control should be listed under subheadings tagged (a), (b) and (c), where (a) represents CKD subjects, (b) represents Hypertensive non-CKD subjects, and (c) represents normotensive non-CKD controls. In their write –up, the author(s) have tagged the hypertensive as controls, rather than subjects. Otherwise, the authors should clarify this confusion on who are the subjects and who are the controls.

6. **Methodology:** The proposed methods for the study are quite satisfactory. On page 7 under BP measurements I suggest that the author(s) should allow time for training the practice parts on getting accustomed to the -ambulatory BP machine and on appropriate response/attitude during the programmed measurements periods to reduce errors.
7. **Stastical Analysis:** This is detailed and satisfactory.
8. **Ethical consideration:**
   - Confidentiality and protection of rights of participants: Well stated and are acceptable.
   - Beneficence: Well stated, and acceptable.
   - Risk to participants: There is going be slight pain to the participants as a result of venipuncture. This was not stated, and should be stated by the investigator(s).
9. **Discussion of Information from the study:** Plans on doing this appropriately should be stated by the investigators.
10. **References supporting the study:** Adequate and relevant. This is satisfactory.

11. **Others:** Some typical, synthase and grammartic errors are noted in the body of the proposal especially on pages 1, 2, 3, 4, 7, 8, 10 and 12. These should be corrected and where necessary further clarifications given.

Conclusion: The proposal corrected based on the above stated recommendations/ suggestions.
